# Coding-Complete Genome Sequences for Two Confirmed Monkeypox Cases in South Africa 2022

**DOI:** 10.1128/mra.00802-22

**Published:** 2022-11-10

**Authors:** Wai Yin Chan, Phillip Senzo Mtshali, Antoinette Grobbelaar, Naazneen Moolla, Thabo Mohale, Michelle Lowe, Morne Du Plessis, Arshad Ismail, Jacqueline Weyer

**Affiliations:** a National Institute for Communicable Diseases, National Health Laboratory Service, Johannesburg, South Africa; b Department of Biochemistry, Genetics and Microbiology, Forestry and Agricultural Biotechnology Institute, University of Pretoria, Pretoria, South Africa; c Department of Medical Virology, Faculty of Health Sciences, University of Pretoria, Tshwane, South Africa; d Department of Biochemistry and Microbiology, Faculty of Science, Engineering and Agriculture, University of Venda, Thohoyandou, South Africa; e Department of Microbiology and Infectious Diseases, Faculty of Health Sciences, University of Witwatersrand, Johannesburg, South Africa; KU Leuven

## Abstract

The coding-complete genome sequences of monkeypox virus (MPXV) were obtained from skin lesion swabs from two human cases detected in South Africa in June 2022. Sequence analyses indicated that the genetic sequences of the viruses associated with these two cases were related most closely to the genetic sequences of other MPXVs reported during the 2022 multicountry outbreak and belong to the monkeypox hMPXV-1 clade (previously West Africa clade) and B.1 lineage.

## ANNOUNCEMENT

The monkeypox virus (MPXV) is a zoonotic virus belonging to the *Orthopoxvirus* genus. The virus is associated with a yet-to-be-determined natural animal host from the Western and Central African regions, with human cases rarely reported (1, 2). MPXV is classified into two clades, namely, I and II (previously known as the Central Africa and West Africa clades, respectively), with 3 sublineages, including Ia, IIa, and IIb (3, 4). Since May 2022, an unprecedented outbreak of monkeypox with human-to-human transmission and multicountry spread has been reported (5), as a result of a new lineage (B.1) which diverged from clade IIb (6).

Here, we report the coding-complete genome sequences of two confirmed monkeypox cases detected in South Africa in June 2022. These sequences were generated using a metagenomics approach. The first case involved a 30-year-old male residing in the Gauteng Province of South Africa (sample reference NICD-SVPL223), and the second case involved a 32-year-old male residing in the Western Cape Province (sample reference NICD-SVPL232). Both cases reported no travel history prior to becoming ill but did report contact with individuals that did travel abroad and may have served as possible sources of exposure.

This study was approved by the Human Research Ethics Committee of the University of the Witwatersrand Johannesburg South Africa (protocol number: M210752). Nucleic acids were isolated from swabs collected from skin lesions using the QIAamp viral RNA mini kit (Qiagen, Germany) according to the manufacturer’s instructions. DNA was quantified with the Qubit double-stranded DNA (dsDNA) high-sensitivity assay (Life Technologies, USA) on the Qubit 3.0 instrument (Life Technologies) according to the manufacturer’s instructions. Shotgun metagenomics sequencing was performed using 11 to 14 ng of the extracted DNA. Multiplexed paired-end libraries were prepared using the Illumina Nextera DNA flex preparation kit followed by sequencing (2 × 150 bp) on a NextSeq 550 instrument (Illumina, Inc., USA). Low-quality and adaptor sequence regions were trimmed using trimGalore v0.6.2 (https://www.bioinformatics.babraham.ac.uk/projects/trim_galore/). Host sequence reads were removed using Bowtie 2 v2.4.2 (7) against a human reference genome (GCA_000001405). The remaining reads were mapped to the reference genome MPXP_UK_P2 (NCBI accession number MT903344) with BWA-MEM v0.7.17 (8) followed by variant calling with LoFreq v2.1.3.1 (9) and bcltools v1.10.2-2. The consensus sequence was generated with iVar v1.0 (10), using the output of the SAMtools v1.15 (11) mpileup-aligned BAM output file with a consensus frequency threshold of 0.6. Gene annotations and open reading frames were predicted using Genome Annotation Transfer Utility (GATU) (12) with MPXP_UK_P2 as the reference. The remaining reads were also subject to *de novo* assembly with SPAdes v3.14.1 (13) and the Galaxy monkeypox workflow (https://galaxyproject.org/projects/mpxv/) (14) as additional verification of the assembly result. Default parameters were used unless otherwise stated.

**FIG 1:**
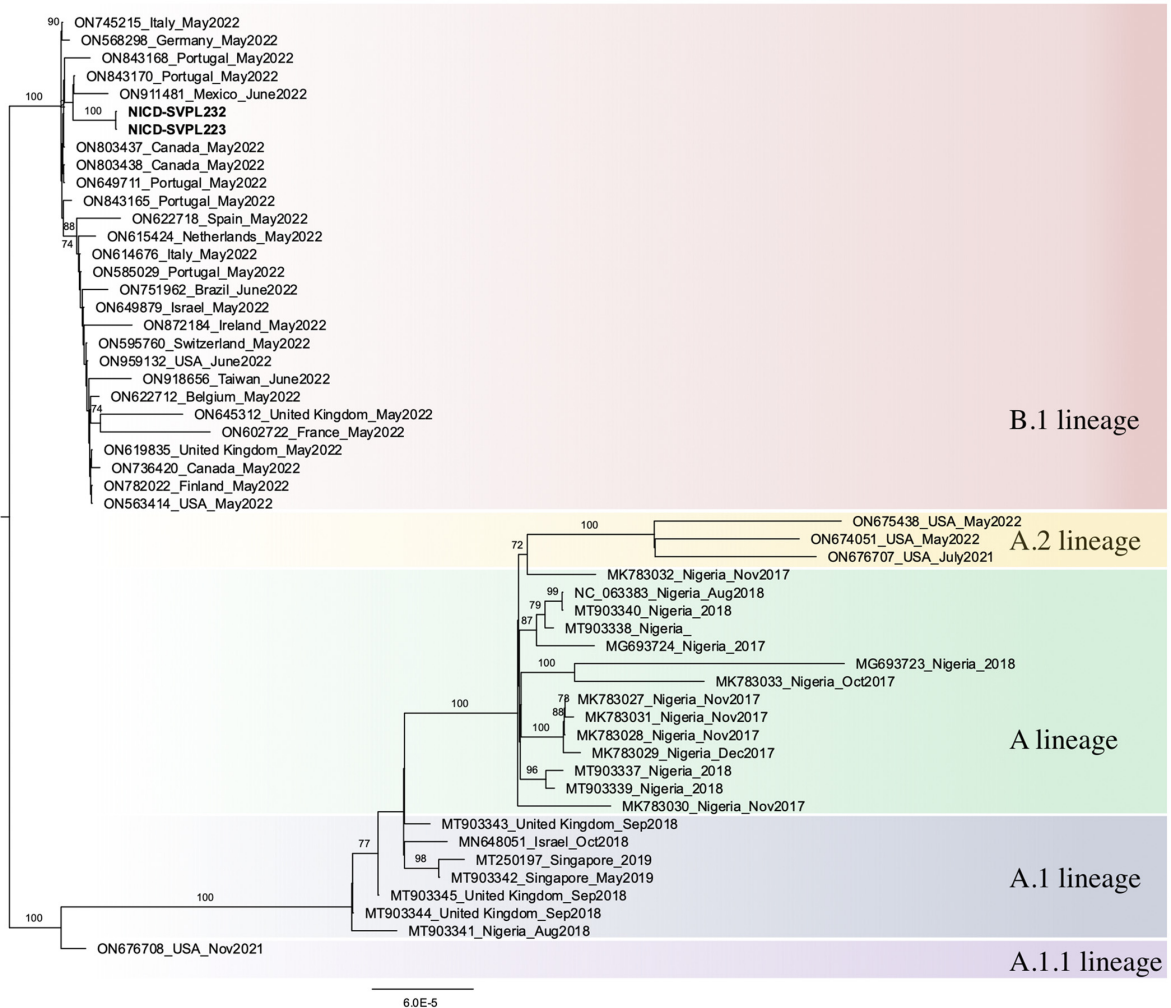
Maximum likelihood tree of the hMPXV-1 clade. Both NICD-SVPL223 and SVPL232 grouped in the hMPXV-1 clade, B.1 lineage. Alignment of the MPXV genomes and the consensus sequences of NICD-SVPL223 and NICD-SVPL232 were generated using MAFFT v7.505 (16) and were curated. The phylogenetics tree was constructed using IQ-TREE2 v2.0.3 (17) using the best-fit model from ModelFinder and a 1,000 ultrafast bootstrap.

The coding-complete genomes consist of double-stranded DNA with a genome size of 197 kb (Table 1) and a genome coverage of 99.9% when aligned to the reference genome sequence. The differences between the two reported assembly sequences to the reference genome are represented by 37 single nucleotide mutations (23 nonsynonymous and 14 synonymous mutations). The commonest nucleotide mutations detected were G > A (23/37), C > T (12/37), GA>AA APOBEC3-like (14/37), and TC>TT (9/37). Publicly available MPXV genome sequences with near-complete to complete status were collected from NCBI (15) for phylogenetic analysis, which indicates that the South African-associated MPXV cases belonged to the B.1 lineage of the hMPXV-1 and that they are related most closely to other MPXV genetic sequences reported during the multicountry outbreak of 2022 (Fig. 1).

**TABLE 1:** Next-generation sequence data mapping statistics of the genomes of MPXV

Sample	Total no. of reads	No. of reads passing QC^*a*^	No. of reads mapped to MPXV reference	Sequencing coverage (×)	Total genome size (bp)	GC content (%)
NICD-SVPL223	169,216,239	169,039,316	560,807	391.433	197,213	32.99
NICD-SVPL232	53,526,611	53,164,490	28,670	20.1	197,126	33

a QC, quality control.

### Data availability.

The genome sequences for NICD-SVPL223 and NICD-SVPL232 were submitted to NCBI GenBank (accession numbers ON918611 and ON927248). Raw sequence data have also been deposited in NCBI (BioProject accession number PRJNA856120 and SRA accession number SRR19995508 and SRR19995509).
